# Elevated Levels of Renalase, the β-NAD(P)H Isomerase, Can Be Used as Risk Factors of Major Adverse Cardiovascular Events and All-Cause Death in Patients with Chronic Kidney Disease

**DOI:** 10.3390/biom11101514

**Published:** 2021-10-14

**Authors:** Wojciech Knop, Natalia Maria Serwin, Elżbieta Cecerska-Heryć, Bartłomiej Grygorcewicz, Barbara Dołęgowska, Aleksandra Gomółka, Magda Wiśniewska, Kazimierz Ciechanowski

**Affiliations:** 1 Clinical Department of Nephrology, Transplantology, and Internal Medicine, Pomeranian Medical University in Szczecin, 70-111 Szczecin, Poland; wojciechknop@gmail.com (W.K.); aemgomolka@gmail.com (A.G.); mwisniewska35@gmail.com (M.W.); kazcie@pum.edu.pl (K.C.); 2 Department of Laboratory Medicine, Pomeranian Medical University in Szczecin, 70-111 Szczecin, Poland; blanka23@pum.edu.pl (E.C.-H.); bartlomiej.grygorcewicz@pum.edu.pl (B.G.); barbara.dolegowska@pum.edu.pl (B.D.)

**Keywords:** renalase, chronic kidney disease, major adverse cardiovascular outcomes

## Abstract

Background: Renalase is an enzyme and a cytokine involved in cell survival. Since its discovery, associations between it and both cardiovascular and kidney disease have been noted. Recognizing this, we conducted a study in which we followed patients with chronic kidney disease. Material and methods: The study involved 90 CKD patients with varying stages of the disease and 30 healthy controls. Renalase was measured with an ELISA kit, and patients were followed-up after a median of 18 months. During the follow-up, we asked about the occurrence of MACE, all-cause mortality and the need for dialysis initiation. Results: In CKD subgroups, RNSL correlated with all-cause death only in the HD group (Rs = 0.49, *p <* 0.01). In the whole CKD population, we found a positive correlation of RNSL concentration and both MACE occurrence (Rs = 0.38, *p <* 0.001) and all-cause death (Rs = 0.34, *p <* 0.005). There was a significant increase in MACE occurrence probability in patients with elevated renalase levels (>25 μg/mL). Conclusions: Elevated renalase levels can be used as a risk factor of MACE in patients with CKD, but its long-term utility needs further research. High renalase levels are a risk factor of death among CKD patients. In HD patients, all deaths were observed among patients with >30 μg/mL; this level could be used as a “red flag” marker in future studies.

## 1. Introduction

Renalase (RNLS) is a small flavoprotein produced mainly by the kidney. However, the latest investigations show that RNLS might be an “organolase”, as the *RNLS* gene is expressed in many other cells and tissues, including the nervous system, endocrinal and digestive tract organs, lungs or heart in humans and some other mammals [1]. RNLS shows both intracellular and extracellular activity. Intracellular RNLS acts as an enzyme that, in the presence of a FAD cofactor, oxidizes 2- and 6- DHNAD(P) to β-NAD(P)+, its biologically active form. This action prevents toxicity resulting from the inhibition of many β-NAD(P)+ dependent enzymes and reactions. Additionally, extracellular renalase and RP-200 and RP-220 peptides, which are fragments of the protein, activate some signaling pathways, including Akt and MAP kinases, therefore promoting cell survival. This activity is mediated by the binding of renalase to its recently discovered receptor–plasma membrane Ca^2+^-ATPase-4b (PMCA4b) [2].

Despite reported discrepancies in observed serum renalase levels in humans, most analyses indicate that serum RNLS levels significantly increase in people with chronic kidney disease (CKD). As in the case of many other markers, this relationship would demonstrate the usefulness of the RNLS concentration assessment in the diagnosis and prognosis of kidney diseases and accompanying disorders. Seeing that CVD is the most significant contributor to mortality of CKD patients, we sought to answer whether RNLS could be a predictive factor for CVD in CKD.

## 2. Materials and Methods

### 2.1. Study Design

One hundred twenty people (aged 40–79) were enrolled into the study. We divided 90 CKD patients into subgroups, each consisting of 30 participants: CKD stage III (CKD III), CKD stage IV (CKD IV), CKD stage 5D who were hemodialyzed (HD) and 30 adults (15 women and 15 men) with no signs of chronic kidney disease (control). Both groups were signed up for the study during treatment at a Nephrology Ward and its Admissions Department, Outpatient Clinic and Dialysis Centre.

### 2.2. Material

To obtain blood serum, blood was drawn into test tubes (S-Monovette, Sarstedt, Germany) and left for 30 min at room temperature. The tubes were then centrifuged (10 min, 1000× *g*, room temperature). The obtained material was then transferred to separate tubes and frozen at −80 °C until renalase levels were measured. eGFR, HDL, LDL and TG levels were assessed in an analytical laboratory as soon as possible after sample collection.

### 2.3. Methods

eGFR was calculated using the CKD-EPI Equation (1), where Scr is serum creatinine (mg/dL), κ is 0.7 for females and 0.9 for males, α is −0.329 for females and −0.411 for males, min indicates the minimum of Scr/κ or 1 and max indicates the maximum of Scr/κ or 1) [3]. Renalase levels were measured in blood serum using an ELISA kit (CloudClone Corp, Houston, TX, USA). Biochemical parameters in serum were evaluated using routine laboratory methods.

(1) $$eGFR=141\times \min{\left(\frac{Scr}{\kappa },1\right)}^{\alpha }\times \;\max{\left(\frac{Scr}{\kappa },1\right)}^{-1.209}\times 0.993^{\mathrm{age}}\times 1.018\;[\mathrm{if}\;\mathrm{female}]\times 1.159\;[\mathrm{if}\;\mathrm{black}]$$

Cardiovascular risk (CV) was calculated using the ASCVD (atherosclerotic cardiovascular risk) algorithm, which includes: age, sex, race, systolic and diastolic blood pressure, total cholesterol and HDL/LDL fractions, diabetes, smoking and hypertension [4]. Participants with acute kidney injury or a history of kidney transplantation were excluded from the study. All patients have given written informed consent.

Follow-up was administered by means of a questionnaire during an outpatient clinic visit, dialysis treatment or a telephone call. Mean follow-up time was 17.3 months. Two patients were lost to follow-up—one patient in control and one in the CKD IV group. In the follow-up, we asked about the first major adverse cardiovascular event (MACE). We defined MACE as an occurrence of either ischemic stroke, acute coronary syndrome, hospitalization due to heart failure or death attributed to a cardiac event. We also looked for all-cause mortality and a need to start renal replacement therapy (RRT).

### 2.4. Statistical Analysis

Obtained data were evaluated using Statistica 13.0 software (StatSoft, Tulsa OK, USA). Since almost all parameters had distributions different than normal, non-parametric tests were used. Differences between two groups (control and CKD) were evaluated using the Mann–Whitney U test. Differences between analyzed parameters in subgroups (control, CKD III, CKD IV, HD) were assessed using Kruskal–Wallis ANOVA with post-hoc Dunn’s test. Correlations were evaluated using Spearman’s Rank Correlation Coefficient. The analysis of the occurrence of MACE and overall survival was performed using the Kaplan–Meier method. The significance level was set at α = 0.05.

## 3. Results

### 3.1. Data Obtained during Recruitment

The study population’s demographic and descriptive data are shown in Table 1 and Table 2 (divided as control and CKD stage-based subgroups) and Table 3 (divided as control and whole CKD group).

When analyzing the control group and CKD-based subgroups, renalase levels were significantly higher in HD than in any other group and the CKD IV group compared to the CKD III group. There was no significant difference in renalase levels between the control group and CKD III and IV groups.

**Figure 1:**
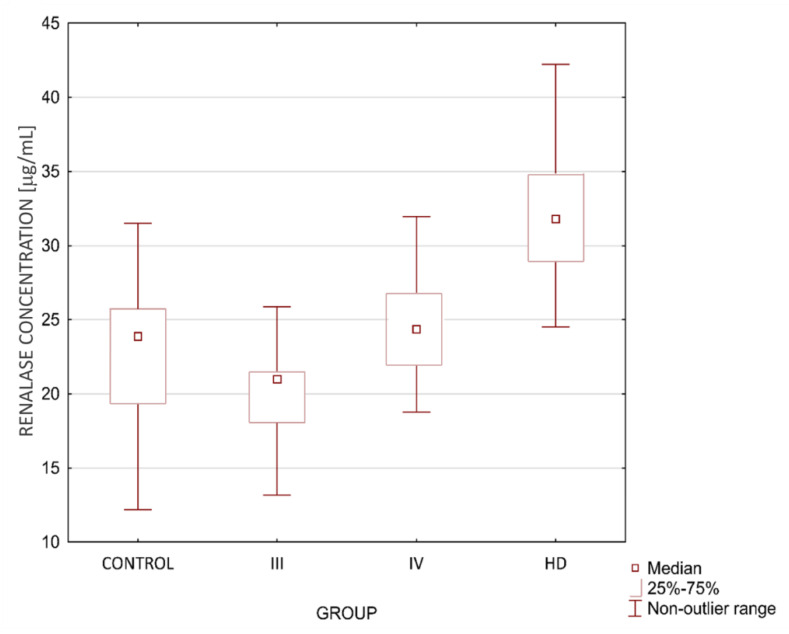
Renalase levels in the studied groups.

Renalase levels in subgroups are shown graphically below in Figure 1.

In the control group, renalase correlated negatively with body weight (Rs = −0.39, *p* < 0.05). There was also a borderline correlation with systolic (Rs = −0.31, *p* = 0.08) and diastolic (Rs = −0.32, *p* = 0.09) blood pressure.

In the CKD III group, renalase did not correlate with any of the analyzed parameters, but in the stage IV CKD group, it correlated again with eGFR (Rs = −0.48, *p* < 0.01) and body weight (Rs = −0.44, *p* < 0.05).

In the HD group, renalase correlated negatively with eGFR (Rs = −0.55, *p* < 0.05) and positively with residual diuresis (Rs = −0.36, *p* < 0.05).

Comparing adults with no kidney disease and the whole CKD group, there was no significant difference in renalase levels, but this difference was at the borderline significance level (*p* = 0.07).

**Figure 2:**
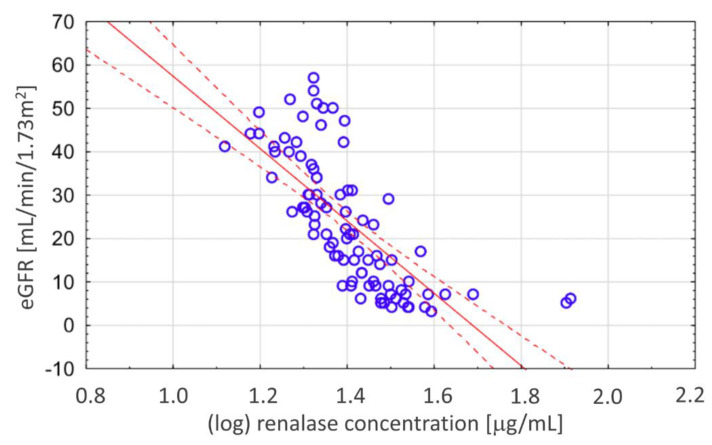
The negative, strong correlation between renalase and eGFR in the CKD group.

In the whole CKD group, we found a strong negative correlation between renalase levels and eGFR (Rs = −0.83, *p* < 0.001), as shown in Figure 2.

When analyzing data from all participants together (control and CKD patients), renalase correlates with diastolic blood pressure (Rs = −0.18, *p* < 0.05), eGFR (Rs = −0.59, *p* < 0.001) and body weight (Rs = −0.32, *p* < 0.001).

### 3.2. Follow-Up Data

During the follow-up period, we recorded 22 MACEs—2 in control, 2 in CKD III, 5 in CKD IV, and 13 in the HD group. There were 11 deaths; none in control and CKD III, four in CKD IV and seven in HD. We observed five HD initiations. There was no RRT other than hemodialysis initiated. Data are shown in Table 4 and Table 5.

Even though statistical analysis showed significant differences in both MACE occurrence and all-cause mortality in all subgroups, the post-hoc analysis did not show specific significance between particular groups. All RRT initiations were observed in the CKD IV group.

Observed time to first MACE was significantly shorter in the HD group than in the CKD IV group. There was no statistical difference between time to all-cause death between groups in which deaths were observed. The median time to RRT initiation was 12 months.

In CKD subgroups, RNLS correlated with all-cause death occurrence in the HD group (Rs = 0.49, *p <* 0.01), but no other significant correlation was found.

When analyzing the whole CKD patient population, we found a positive correlation of RNLS concentration and MACE occurrence (Rs = 0.38, *p* < 0.001) and a negative correlation with time to first MACE occurrence (Rs = −0.52, *p* < 0.05). The correlation of RNLS and MACE occurrence was stronger than that of the algorithm derived, calculated risk (Rs = 0.25, *p* < 0.05). All-cause death also correlated positively with renalase concertation (Rs = 0.34, *p* < 0.005).

Data pooled from all participants (both with and without CKD) concerning endpoints have shown a correlation between RNLS and MACE occurrence (Rs = 0.33, *p* < 0.001), time to MACE (Rs = −0.50, *p* < 0.05) and all-cause death (Rs = 0.31, *p* < 0.001). This conclusion takes into account that a small number of endpoints were observed in the control group.

### 3.3. Analysis of Survival and MACE Occurrence

Based on obtained data, specifically, renalase levels and correlations between renalase and occurrence of MACE and all-cause death in the CKD group, we first divided the CKD group into two subgroups with RNLS levels higher and lower than 25 µg/mL.

**Figure 3:**
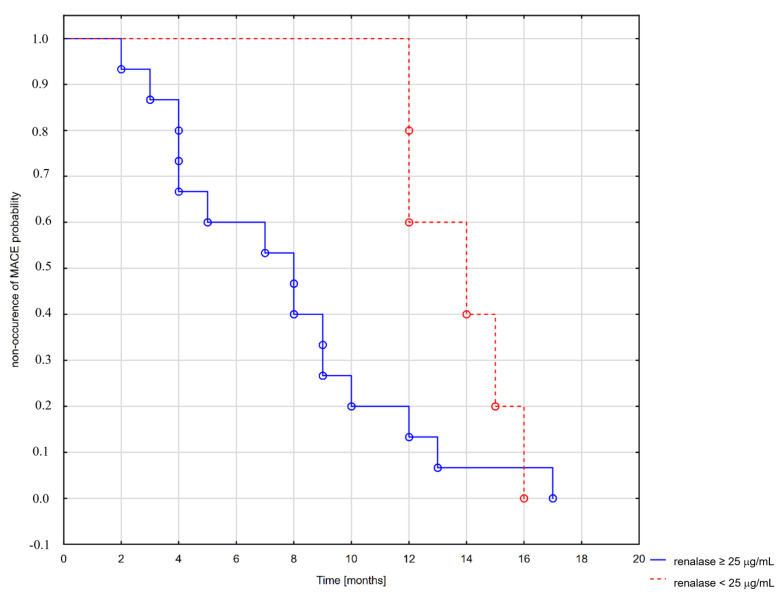
Kaplan Meier curve for MACE in groups with low and elevated renalase levels.

MACE occurred in 20 patients with CKD: 11 with elevated renalase levels (>25 µg/mL) and 9 with lower renalase levels (<25 µg/mL). A Mantel–Cox test *p <* 0.01 showed a significant difference in MACE occurrence between analyzed groups, the odds of which were significantly higher in the group with elevated renalase levels, as shown in Figure 3.

There was no difference in the occurrence of death between subgroups divided basing on the 25 µg/mL threshold.

**Figure 4:**
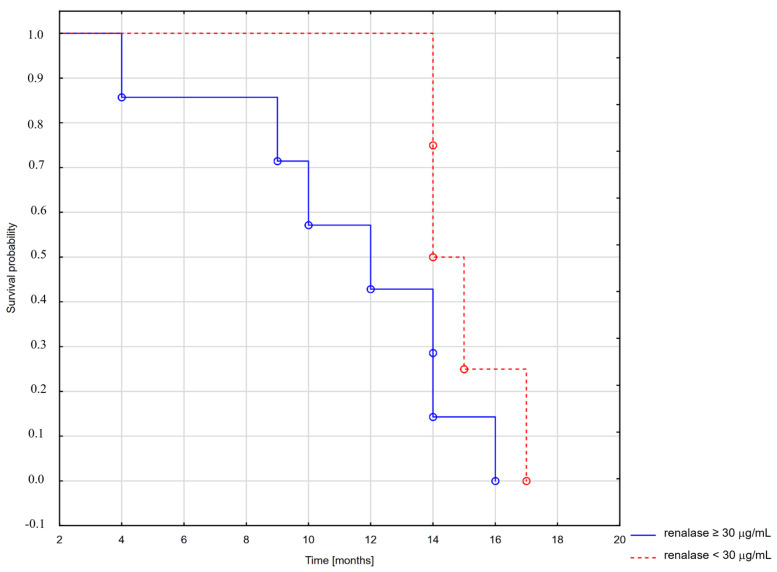
Kaplan–Meier curve for all-cause death in groups with low and high renalase levels.

When a higher threshold was assumed, and patients with CKD were divided into groups with levels of RNLS higher or lower than 30 µg/mL, not only did the higher probability of MACE remain significant, but a higher probability of all-cause death was also observed (but without statistical significance in log-rank test: *p =* 0.09), as presented in Figure 4.

In the HD group, in which a significant correlation between RNSL levels and all-cause death was observed, a Kaplan–Meier analysis of survival was not possible as the deaths were observed only in patients with renalase concentration higher than 30 µg/mL. At the same time, there was no significant difference in MACE occurrence in this subgroup.

## 4. Discussion

CKD significantly contributes globally to both morbidity and mortality. Exact numbers are unknown, but screening programs in high-income countries have shown that more than 10% of adults have markers of kidney disease. The global burden of CKD in 2017 was estimated at 6975 million cases. Even though primary causes of CKD vary in different populations, the most common causes are hypertension and diabetes [5]. In Poland, an estimated 4.2 million people have CKD, and about 6500 yearly develop ESRD and require RRT [6]. The lives of patients afflicted with CKD, whether they deal with early-stage disease or end-stage renal disease (ESRD), are associated with high morbidity and increased healthcare utilization [7].

Patients with CKD have an established, higher CVD-related morbidity and mortality, and multiple analyses of studies have shown that the presence of CKD is independently associated with cardiovascular events, especially in groups with preexisting known risk factors [8,9,10]. The exponential increase in risk for cardiovascular mortality risk at low eGFR was shown in a large meta-analysis comprising ten cohorts with 266,975 patients. Hazard ratios at eGFRs of 60, 45 and 15 mL/min per 1.73 m^2^ were 1.03, 1.38 and 3.11, respectively, compared to an eGFR of 95, after adjustment for albuminuria and cardiovascular risk factors [11]. A total of 7.6% of all CVD deaths (1.4 million globally in 2017) could be attributed to impaired kidney function [12].

CVD burden is also qualitatively different in CKD patients than those without renal pathology. Aside from atherosclerosis-related diseases (coronary artery disease, ischemic stroke and peripheral artery disease), other cardiac events become more and more prevalent as kidney function declines. Non-atherosclerotic CVD, arrhythmias, sudden cardiac deaths, arterial calcifications, valve calcifications and haemorrhagic strokes may be caused by uraemia, CKD-MBD, LVH or be dialysis-related events [13]. A growing trend of non-ischemic CVD, to which CKD undoubtedly contributes, has been observed in developed countries [14].

Standard risk factors and assessment tools underperform in CKD patients, as their predictive risk is much lower than observed CVD events [15]. Moreover, we do not have particularly well-designed or widely used recalibration tools, which should at least account for eGFR and albuminuria [10]. Standard and widespread CVD risk assessment tools name CKD as an additional factor to be taken into consideration but do not clearly stratify the eventual risk. CVD risk equations can be of great importance to both patients and clinicians during decision-making. Even the classic symptoms of acute myocardial infarction are reported far less often by CKD patients than people without kidney pathology [13]. Risk-aware and evidence-based medical interventions are needed as CKD prevalence continues to grow. Early action can delay disease progression and mitigate the fact that patients with ESRD consume a disproportionate share of health care resources.

Renalase has been linked to CKD and CVD in numerous studies. In a seminal paper describing renalase discovery, Xu et al. postulated a link to the cardiovascular system [16]; unfortunately, the enzymatic function of catecholamine degradation primarily proposed in the study, directly linking renalase to CVD, was disputed [17,18,19,20,21] and eventually refuted [22]. Different enzymatic activity was observed and described; renalase was found to be an oxidase/isomerase, using molecular oxygen to convert α-NAD(P)H into β-NAD(P)+, with hydrogen peroxide as a reaction byproduct [23], which also turned out to be incorrect [24]. Eventually, renalase has come to be described as an isomerase catalyzing rapid oxidation of 6DHNAD(P) and 2DHNAD(P) (which are inhibitory to metabolism dehydrogenases) to their active form—β-NAD(P)+ [22].

Additionally, since 2014, renalase’s other function has been observed—that of a cytokine. It was found that a part of renalase protein – RP 220 (described previously [25], containing amino acids 220–239) as well as its variations RP-224 (amino acid 224–233) and RP-H220 (histidine-tagged RP 2020) – improved HK-2 cell line survival when treated with cisplatin, independently of amine oxidase activity by activation of the AKT and MAPK signaling pathways [26]. A receptor for renalase was found a year later. In 2015, PMCA4b (plasma membrane ATPase) was discovered to bind with renalase and activate the aforementioned signaling pathways [27], thus rekindling the search for the causative interplay of renalase and CVD, as PMCA4b is connected to both cardiac hypertrophy [28] and hypertension [29]. Aside from catalytic and cytokine mechanisms, yet another metabolic role came to light through further research in 2019: data collected in models of liver injury indicated that renalase might activate SIRT1 by elevating NAD+ levels [30].

Renalase affects redox balance in all functions and is beneficial in states of ischemia and/or reperfusion injury.

Its role in kidney disease has been shown in animal model interventional studies. Contrast-induced nephropathy in rat models was mitigated by supplying exogenous renalase by reducing oxidative stress, among other influences [31]. Renalase has also been found to reduce cisplatin-induced acute kidney injury by decreasing mitochondrial fission and reactive oxygen production [30].

Heart disease is also linked to redox state [32]. As such, studies have shown associations between higher renalase levels and unstable angina pectoris [33], lower ejection fraction in heart failure patients [34,35] and coronary microvascular dysfunction diagnosed with Rb-82 PET/CT imaging [36]. When performed in patients with stable angina, Percutaneous Coronary Intervention decreased serum renalase levels within a few days [37]. Extensive reviews of other observed relationships can be found elsewhere [38,39,40].

Even though we do not fully understand renalase’s mechanism of action, as paradigm shifts seem to occur every few years, renalase displays a wide range of correlations and effects in CVD and both chronic kidney disease and acute kidney injury, which have been found in observational and interventional studies.

Other studies have also tried to assess renalase levels as a predictive factor. A study by Gluba-Brzózka et al. [41] positively correlated renalase with worsening CKD stages, as did markers (both biochemical and functional) of cardiovascular risk, but no observational follow-up was mentioned. Chang et al. [42] conducted a study in which patients (all male) were observed up to 10 years. A statistically significant correlation was found between renalase levels at baseline and both 1- and 5-year all-cause mortality. No correlation was found at 10 years. Using data from a Korean K-STAR cohort, Na et al. [43] observed higher all-cause mortality but not more frequent MACE in CKD patients with higher renalase levels. Another group studied patients recruited during scheduled PCI. In the group characterized by higher renalase levels, more endpoints (comprising myocardial infarction, stroke or death) were recorded during follow-up (median follow-up time lasted 4.1 years) [37]. In yet another recent study by Cerqueira et al., 40 pre-dialysis CKD patients were followed up for a mean period of 65 months. RNLS was associated with CKD progression, hospitalizations and all-cause mortality, but not with MACE occurrence [44]. Our study results are in line with these findings. Renalase levels found in studies mentioned in this paragraph are summarized in Table 6.

## 5. Conclusions

Loss of kidney function is accompanied by an accumulation of renalase in blood serum.

- Elevated renalase levels (>25 µL/mL) are a risk factor of MACE in patients with chronic kidney disease, but its long-term utility needs further research.
- High renalase levels (>30 µg/mL) can be a risk factor of death among CKD patients.
- In HD patients, all deaths were observed among patients with a renalase serum concentration greater than 30 μg/mL. Further research could show a possible “threshold” level, prompting an evaluation of patients to either intensify treatment or start discussing palliative measures.

## Figures and Tables

**Table 1:** Descriptive data of the subgroups together with statistical evaluation (Kruskal-Wallis ANOVA). Data are shown as mean ± SD and median (lower quartile–upper quartile).

Parameter	Control n = 30		CKD III n = 30		CKD IV n = 30		HD n = 30		*p*-Value
	Mean ± SD	Median (LQ-UQ)	Mean ± SD	Median (LQ-UQ)	Mean ± SD	Median (LQ-UQ)	Mean ± SD	Median (LQ-UQ)	
Age (Years)	57 ± 11 ^1,2^	56 (45– 67)	68 ± 8 ^1^	69 (62–74)	66 ± 11 ^2^	67 (60–75)	64 ± 11	65 (60–74)	0.0028
Systolic BP (mmHg)	131 ± 21 ^1^	125 (120–140)	134 ± 12	132 (130–140)	140 ± 18 ^1^	140 (130–160)	140 ± 21	140 (130–150)	0.0216
Diastolic BP (mmHg)	80 ± 11	80 (70–85)	79 ± 9	80 (75–85)	80 ± 11	80 (75–90)	77 ± 12	80 (7–80)	0.44
eGFR	85 ± 13	85 (72–91)	41 ± 8	41 (34–48)	22 ± 5	21 (17–26)	7 ± 3	7 (5–9)	<0.001
Body Weight (kg)	83 ± 20	80 (70–94)	84 ± 15 ^1^	84 (75–92)	82 ± 16	80 (71–92)	72 ± 16 ^1^	72 (64–83)	0.0286
Height (cm)	171 ± 8	170 (164–176)	169 ± 8	169 (160–176)	167 ± 9	167 (160–175)	168 ± 11	165 (158–178)	0.50
LDL (mg/dL)	122 ± 35	124 (97–148)	119 ± 47	128 (79–156)	119 ± 52	106 (82–151)	110 ± 46	105 (70–136)	0.55
HDL (mg/dL)	55 ± 16	52 (45–61)	50 ± 17	48 (39–61)	52 ± 27	41 (37–65)	49 ± 11	48 (42–54)	0.37
TC (mg/dL)	174 ± 48	187 (130–208)	187 ± 47	188 (151–232)	191 ± 64	84 (144–247)	179 ± 57	162 (136–203)	0.66
TG (mg/dL)	167 ± 91	161 (105–214)	168 ± 102	135 (96–208)	202 ± 26	171 (116–246)	159 ± 81	145 (103–196)	0.60
Renalase (µg /mL)	21.8 ± 9.2 ^1^	23.9 (19.4–25.9)	20.2 ± 3.1 ^2,4^	21.0 (18–21.5)	24.9 ± 4.1 ^3,4^	24.6 (2.0–26.8)	35.6 ± 13.5 ^1,2,3^	31.79 (28.9–34.9)	*p <* 0.01
CV Risk Prediction Score (%)	11.3 ± 12.1 ^1,2^	6.1 (1.4–19.8)	24.0 ± 14.6 ^1^	24.0 (11.3–31.5)	25.0 ± 17.2 ^2^	22.7 (9.8–39.9)	20 ± 17	17 (6–31)	0.0012

Abbreviations: BP—blood pressure; eGFR—estimated glomerular filtration rate; LDL—low-density cholesterol; HDL—high-density cholesterol; TC—total cholesterol; TG—triglycerides; CV—Cardiovascular. ^1,2,3,4^—the result of the post-hoc analysis showing differences between the indicated groups.

**Table 2:** Qualitative risk factors occurrence in analyzed subgroups (*p*-values derived from Kruskal–Wallis ANOVA analysis).

Parameter	Control n = 30	CKD III n = 30	CKD IV n = 30	HD n = 30	*p*-Value
Hypertension n = 95	12 ^1,2,3^	28 ^1^	29 ^2^	26 ^3^	<0.05
Diabetes n = 37	7	10	11	9	>0.05
Smoking n = 21	7	6	5	3	>0.05
No Residual Diuresis n = 6	0	0	0	6	<0.05

Arterial hypertension was significantly more common in all CKD subgroups than in control. There was no statistical difference between diagnosed diabetes and active smoking in all groups. ^1,2,3^—the result of the post-hoc analysis showing differences between the indicated groups.

**Table 3:** Demographic and descriptive data and statistical evaluation between control and the whole CKD group (Mann–Whitney U test).

Parameter	Control		CKD		*p*-Value
	Mean ± SD	Median (LQ–UQ)	Mean ± SD	Median (LQ-UQ)	
Age (Years)	57 ± 11	56 (45–67)	66 ± 7	67 (60–74)	<0.001
Systolic BP (mmHg)	131 ± 21	125 (120–140)	135 ± 18	132 (120–140)	0.01
Diastolic BP (mmHg)	80 ± 11	80 (70–85)	80 ± 10	80 (70–90)	0.94
eGFR	85 ± 13	85 (72–91)	49 ± 28	41 (26–72)	<0.001
Body Weight (kg)	83 ± 20	80 (70–94)	83 ± 17	80 (72–92)	0.74
Height (cm)	171 ± 8	170 (164–176)	169 ± 9	170 (161–176)	0.20
LDL (mg/dL)	122 ± 35	124 (97–148)	120 ± 44	123 (84–151)	0.34
HDL (mg/dL)	55 ± 16	52 (45–61)	52 ± 20	50 (40–63)	0.07
TC (mg/dL)	174 ± 48	187 (130–208)	184 ± 53	184 (144–217)	0.63
TG (mg/dL)	167 ± 91	161 (105–214)	179 ± 107	161 106–214)	0.98
Renalase (µg/mL)	21.8 ± 9.2	23.9 (19.4–25.9)	22.3 ± 6.3	22.5 (19.9–25.1)	0.07
CV risk (%)	11.3 ± 12.1	6.1 (1.4–19.8)	22.8 ± 16.3	20.8 (9.4–31.5)	<0.001

Abbreviations: BP—blood pressure; eGFR—estimated glomerular filtration rate; LDL—low-density cholesterol; HDL—high-density cholesterol; TC—total cholesterol; TG—triglycerides; CV—Cardiovascular.

**Table 4:** Qualitative data on recorded endpoints; data are shown as number of individuals (*p*-values derived from Kruskal–Wallis ANOVA analysis).

Parameter	CONTROL n = 29	CKD III n = 30	CKD IV n = 29	HD n = 30	*p*-Value
MACE n = 22	2	2	5	13	0.0006
Deaths n = 11	0	0	4	7	0.0035
Dialysis Initiation n = 5	0	0	5	N/A	N/A

Abbreviations: MACE—major adverse cardiovascular events, CKD—chronic kidney disease.

**Table 5:** Quantitative data on recorded endpoints with statistical evaluation (Kruskal-Wallis ANOVA). Data are shown as mean ± SD and median (lower quartile—upper quartile).

Parameter	CONTROL n = 29		CKD III n = 30		CKD IV n = 29		HD n = 30		*p*-Value
	Mean ± SD	Median (LQ-UQ)	Mean ± SD	Median (LQ-UQ)	Mean ± SD	Median (LQ-UQ)	Mean ± SD	Median (LQ-UQ)	
Time to MACE (Months)	6 ± 5	6 (3–10)	9 ± 3	9 (7–12)	14 ± 2 ^1^	14 (13–15)	7 ± 4 ^1^	8 (4–9)	0.028
Time to Death (Months)	N/A	N/A	N/A	N/A	15 ± 4.5	14.5 (14–16)	11 ± 4	12 (9–14)	>0.05
Time to HD Initiation (Months)	N/A	N/A	N/A	N/A	10 ± 4.5	12 (11–12)	N/A	N/A	N/A

Abbreviations: MACE—major adverse cardiovascular events; CKD—chronic kidney disease; HD—hemodialysis. ^1^—the result of the post-hoc analysis showing differences between the indicated groups.

**Table 6:** Renalase levels in various groups in studies by other authors.

Publication	Group	Level	Group	Level
Markers of Increased Cardiovascular Risk in Patients with Chronic Kidney Disease [41]	Control Group n = 45	mean: 251.0 ± 157.0 ng/mL	CKD all stages n = 132	mean: 316.1 ± 155.3
Identification of Two Forms of Human Plasma Renalase, and Their Association with All-Cause Mortality [42]	Normal renal function n = 10	mean: 20.39 ± 7.70 µg/mL	All patients n = 267	mean: 18.8 ± 8.5 µg/mL
Circulating renalase predicts all-cause mortality and renal outcomes in patients with advanced chronic kidney disease [43]	Control group n = 16	mean: 28.2 ± 5.1 µg/mL	CKD patients n = 383	mean: 75.8 ± 34.8 µg/mL
Serum Renalase Levels Are Predicted by Brain-Derived Neurotrophic Factor and Associated with Cardiovascular Events and Mortality after Percutaneous Coronary Intervention [37]	Before percutaneous coronary intervention n = 152	mean: 47.5 ± 17.3 ng/mL	After percutaneous coronary intervention n = 152	mean: 35.9 ± 11.3 ng/mL
Circulating Renalase as Predictor of Renal and Cardiovascular Outcomes in Pre-Dialysis CKD Patients: A 5-Year Prospective Cohort Study [44]	CKD stages 1 and 2 n = 17	mean: 42.03 µg/mL	CKD stages 4 and 5 n = 14	mean: 83.53 µg/mL

Abbreviations: CKD—chronic kidney disease.
